# Layer-Specific Proteomic Profiling of the Human Cornea Reveals Insights Into Structure and Biological Function

**DOI:** 10.1167/iovs.67.1.44

**Published:** 2026-01-21

**Authors:** Hauke M. Schadwinkel, Paula Nissen, Manuela Moritz, Stephan J. Linke, Andrea Hassenstein, Larissa Lohner, Olaf Hellwinkel, Hartmut Schlüter, Martin S. Spitzer, Maria Steuernagel, Jan Hahn

**Affiliations:** 1 Department of Ophthalmology, University Medical Center Hamburg-Eppendorf, Hamburg, Germany; 2 Core Facility Mass Spectrometric Proteomics, Center for Diagnostics, University Medical Center Hamburg-Eppendorf, Hamburg, Germany; 3 Section Mass Spectrometry and Proteomics, Center for Diagnostics, University Medical Center Hamburg-Eppendorf, Hamburg, Germany; 4 Zentrumsehstärke, Augenarztpraxis am UKE, Hamburg, Germany; 5 Institute of Legal Medicine, Center for Diagnostics, University Medical Center Hamburg-Eppendorf, Hamburg, Germany; 6 Mildred Scheel Cancer Career Center Hamburg, University Medical Center Hamburg-Eppendorf, Hamburg, Germany

**Keywords:** spatial proteomics, human cornea, near-infrared femtosecond laser ablation (NIRL), corneal biology

## Abstract

**Purpose:**

Clinical proteomics enhances our understanding of biological functions and pathological processes. Since the localization of various protein clusters within the cornea is still unknown, a spatial model of the human corneal proteome was established.

**Methods:**

In this study, successive layers of corneal tissue from five human corneas were ablated using a nanosecond mid-infrared laser, with ablation depth verified by optical coherence tomography. Each layer was analyzed by LC-MS/MS-based quantitative proteomics to generate a spatial map of the corneal proteome.

**Results:**

A total of 4,454 proteins were identified. A clear distinction between proteome clusters reflecting the tissue layers within the cornea was observed. Various biological processes were localized in the individual segments of the cornea. Increased abundances of metabolic proteins in the epithelium reflected high metabolic activity and regeneration processes. The endothelium was characterized by high energy demand and increased levels of transmembrane proteins. Notably, the stroma exhibited significantly higher abundances of immune-related proteins. A distinct proteomic profile was also observed in the subepithelial region, which is clinically involved in corneal wound healing.

**Conclusions:**

These results highlight the proteomic differentiation and functional specialization of individual corneal layers and provide insights into key biological processes, including immune responses, wound healing, and corneal homeostasis. Additionally, these findings hold the potential to be contrasted with pathological conditions and to trace the morphological localization of pharmacological target molecules.

Besides its crucial role in light refraction, which contributes over 70% of the eye's total refractive power, the cornea also performs several other important ocular functions including protection against biological and chemical damage, as well as providing biomechanical stability and structural resilience.^1^^,^^2^ Corneal diseases such as infections, Fuchs' endothelial dystrophy, traumatic injuries or keratoconus are a leading cause of blindness worldwide and affect millions of individuals.^3^^–^^6^ Despite existing pharmacological and surgical treatments, a deeper functional understanding of corneal biology and disease mechanisms is essential for developing more effective therapeutic strategies.^7^

The cornea consists of five main layers, with each contributing to its optical and biological functions. While the epithelium primarily serves as a protective barrier, the stroma consists of a collagen network with few keratocytes and accounts for most of the corneal thickness. The endothelium maintains stromal dehydration and transparency through its active pump function.^8^ The structural stratification of the cornea provides a rationale for spatially resolved proteomic studies.

Several studies have examined the proteome of the human cornea.^9^^–^^17^ However, most of these studies homogenized the entire corneal tissue to gain insights into intracorneal proteomic processes.^12^^,^^18^ When comparing the proteomics studies performed to date, the highest proteome coverage corresponding to 4,824 proteins was achieved using mechanical homogenization of whole corneal tissue.^14^ Although these findings have expanded our knowledge of corneal physiological mechanisms and pathologies like keratoconus,^15^^,^^16^ analyzing the cornea in its entirety fails to capture the distinct molecular signatures of its individual layers.

In some studies, the tissue was manually dissected and the distinct layers of the cornea, such as the epithelium, stroma or endothelium, were separated and analyzed.^17^^,^^19^ Procedures separating the layers were also used to compare the proteome of corneal pathologies such as keratoconus or Fuchs' endothelial dystrophy with healthy corneas.^20^^–^^22^ However, these methods do not provide the ability to visualize or trace the morphological localization of various proteins or biological processes in the whole cornea, nor can they detect proteomic variations within individual regions of a single anatomical layer such as the stroma.

In addition, proteomic analysis results can be altered by the mechanical homogenization of the tissue^23^^–^^25^ and the large amount of collagen within the cornea and especially in the stroma may dilute lower abundance proteins.^19^ Ablation of the tissue by laser can offer advantages over mechanical homogenization. In the recent years, a new laser ablation technology has emerged, which enables spatial proteomics by tissue sampling followed by conventional bottom-up proteomics workflows.^25^^–^^27^

In this study, we used nanosecond mid-infrared laser (NIRL) layer-by-layer ablation on consecutive layers of corneal tissue, combined with optical coherence tomography (OCT) for visualization of layer thickness. This sampling directly from intact fresh-frozen tissue with three-dimensional resolution followed by LC-MS/MS-based differential quantitative proteomics provided spatial resolution of the human corneal proteome. This method provides layer-specific insights into corneal biology and clinical processes, clarifies associated signaling pathways, and allows mapping of potential pharmacological targets.

## Methods

### Corneal Samples Preparation

The corneal tissue used in this study was obtained from donor eyes from the corneal tissue bank at the Institute of Legal Medicine in Hamburg after appropriate medical information regarding pre-existing conditions and exclusion criteria and consent of the next of kin. The corneas were obtained from five donors aged 68 to 85 years without corneal diseases (two male and three female donors) and were harvested within the first 72 hours postmortem. The corneas were unsuitable for transplantation, as post-harvesting assessment revealed systemic donor diseases contraindicating their use. Exclusion criteria for corneal donation included rapidly progressing dementia, and insufficient blood volume for serological testing required to exclude systemic infectious diseases. The corneas were examined with a hand-held slit lamp and selected only if they were transparent and showed no morphological or structural abnormalities.

The corneas were standardly harvested as sclerocorneal slices after multiple disinfections under sterile conditions using a trephine. They were then transported in Tissue C medium (Alchimia, Ponte San Nicolò, Italy) and processed immediately. The medium was completely removed from the corneas. The sclerocorneal slices were cut into small pieces under the microscope using tweezers and a scalpel without touching the central area of each sample. The samples were stored at −80°C until further processing with the laser ablation setup, followed by mass spectrometric analyses.

### Laser Ablation Setup and Ablation Procedure Parameters

**Figure 1.**
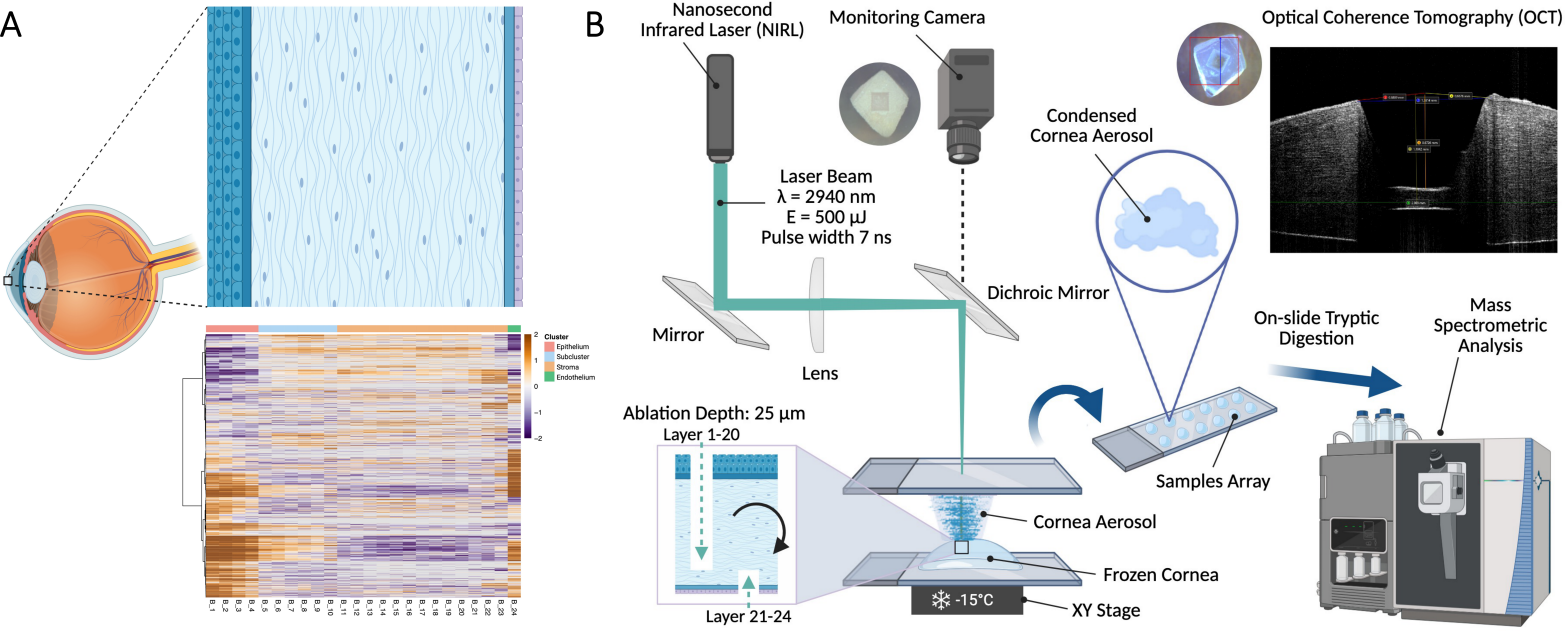
**(A)** Schematic overview of the human cornea and example proteomic heatmap, **(B)** Experimental setup for spatial proteomic analysis of the human cornea combined with OCT visualization. Created in BioRender by H. Schadwinkel (2025).

For the layer-wise tissue sampling, the laser ablation setup and workflow from Figure 1B was used. The basic laser system was already described in Navolić et al.,^27^ but here we used sample scanning with a motorized XY stage (built from two MLT25; Newport Corporation, Irvine, CA, USA), where the controller (XPS-RLD4; Newport Corporation) with the driver cards (XPS-DRV11; Newport Corporation) triggered the laser emission with the maximum repetition rate of 20 Hz. The ablation pattern was programmed on the controller and consists of 10 × 10 applied single laser shots, forming a layer with the dimensions of 1,000 × 1,000 × 25 µm³ (volume of 25 µL), which was determined by three-dimensional imaging with OCT. We applied a layer-by-layer laser ablation targeting the central and paracentral pieces of the cornea, starting from the anterior side. Each sampled layer was collected on a separate well of a polytetrafluoroethylene-coated objective glass slide for potential downstream proteomic analysis. This sampling procedure was then repeated from the posterior side with a transversal shift up to a depth with a specific overlap. The specific parameters for the ablation patterns were acquired through previous experiments including thickness and ablation depth measurements using OCT.

A total of 24 samples were taken from each cornea, following a specific pattern: the first eight samples were collected through consecutive ablations to achieve detailed resolution of the epithelial and subepithelial layers. For the subsequent twelve samples and in the region of the stroma, every fourth ablation was used. The final four samples were obtained by rotating the sample to maximize the resolution of the endothelial side, with four samples taken consecutively from the endothelial layer. The samples were labeled according to their anatomical order, starting from the epithelial side with Sample 1 and progressing to Sample 24, which corresponded to the endothelial side. Because of technical issues, we could just use 22 samples from individual A for further analysis (samples 3–24).

### Sample Processing

The condensed aerosol was resuspended in 10 µL of 0.01% n-dodecyl-β-D-maltoside in 25 mM ammonium bicarbonate. Afterwards, the PTFE-coated slide was placed on a heating block and the samples were heated for five minutes at 95°C. During the heating step, aliquots of water (LC-MS grade) were added to keep the samples in solution. Trypsin 20 ng was added to the samples before incubating overnight at 37°C. During that time the samples were stored in a humidity chamber. Finally, the sample slides were dried in a vacuum centrifuge.

### LC-MS/MS

Tryptic peptides were resuspended in 10 µL 0.1% formic acid in water. Chromatographic separation of peptides was achieved with a two-buffer system (buffer A: H2O with 0.1% FA, buffer B: 80/20 (v/v) ACN/H2O with 0.1% FA) on a UHPLC (Vanquish neo UHPLC system; Thermo Fisher Scientific, Waltham, MA, USA). Attached to the UHPLC was a peptide trap (100 µm × 20 mm, 100 Å pore size, 5 µm particle size, C18; Thermo Fisher Scientific) for online desalting and purification, followed by a 25 cm C18 reversed-phase column (75 µm × 250 mm, 130 Å pore size, 1.7 µm particle size, peptide BEH C18, nanoEase; Waters Corporation, Milford, MA, USA). Peptides were separated using a 60 minutes method with linearly increasing concentration of buffer B from 2.5% to 37.5% over 50 minutes.

MS/MS measurements were performed on a quadrupole-orbitrap hybrid mass spectrometer (Exploris 480; Thermo Fisher Scientific). Eluting peptides were ionized using a nano-electrospray ionization source with a spray voltage of 1,800 V and analyzed in data-dependent acquisition (DDA) mode. For each MS1 scan, ions were accumulated for a maximum of 25 ms or until a charge density of 3 × 10^6^ ions (AGC target) was reached. Fourier-transformation based mass analysis of the data from the orbitrap mass analyzer was performed covering a mass range of m/z 350 – 1,400 with a resolution of 60,000 at m/z 200. Peptides responsible for the 20 highest signal intensities per precursor scan with an intensity threshold of 8 × 10^3^ and charge state from +2 to +6 were isolated within an isolation window of m/z 2 and fragmented with a normalized collision energy of 30% using higher energy collisional dissociation (HCD). MS2 scanning was performed, covering a mass range starting at m/z 120 and accumulated for 50 ms or to an AGC target of 1 × 10^5^ at a resolution of 15,000 at m/z 200. Already fragmented peptides were excluded for 30 seconds.

### Data Analysis

LC-MS/MS data were searched with the Sequest algorithm integrated into the Proteome Discoverer software (v3.1.0.638; Thermo Fisher Scientific) against a reviewed human Swissprot database, obtained in April 2021, containing 20,365 entries. The oxidation of methionine, and pyro-glutamate formation at glutamine residues at the peptide N-terminus, as well as the acetylation and methionine loss of the protein N-terminus were allowed as variable modifications. A maximum number of two missing tryptic cleavages was set. Peptides between 6 and 144 amino acids were considered. A strict cutoff (FDR < 0.01) was set for peptide and protein identification. Quantification was performed using the Minora Algorithm, implemented in Proteome Discoverer.

### Statistics

Basic statistical analysis including basic transformation, normalization and testing steps were performed with Perseus software (Max Planck Institute for Biochemistry, v. 2.0.11 and 2.0.10). The obtained quantitative relative protein abundances were log_2_-transformed and column-median normalized over all proteins of one sample. Proteomic data were filtered for proteins present in at least three samples in total. Based on the Pearson correlation coefficient between the sample, the geometrical side of ablation and the histology, clusters were defined within each individual's sample. Student's *t*-testing was performed between each defined cluster and all other layers. All proteins reaching a *q*-value < 0.05 and with a twofold change were considered significantly different. Gene set enrichment analysis (GSEA) was performed with the t-test results using the clusterProfiler package (version 4.14.0) in the R software environment (version 4.4.0) and the gene ontology (GO) terms in biological process (BP), molecular functions (MF), and cellular component (CC).^28^ Data visualization and statistical analysis were performed using R (v4.4.0) and relevant packages including ggplot2, clusterProfiler, and mixOmics. Full details are available in the Supplement.

### Clustering of Different Corneal Regions

To account for inter-individual differences, each individual's corneal layers were analyzed separately based on their proteomic profiles. By using Pearson correlation, layer thickness in OCT, experimental set-up as well as anatomy and histology of the examined corneal samples, four different clusters could be defined. Nonlinear-iterative-squares PCA was used to visualize the proteomic separation of clusters in each individual. The clusters were labeled according to their anatomical localization (Cluster 1 as “Epithelium”, Cluster 2 as “Sublayer”, Cluster 3 as “Stroma”, Cluster 4 as “Endothelium”). The clustering reflects subtle variations in layer thickness among different corneas.

One representative dataset (individual C) was selected based on optimal layer resolution and used as a reference to standardize cluster definitions across the remaining individuals. Correlation-based clustering was then applied to the combined dataset.

## Results

The proteomic analysis of five different corneal samples with consecutive NIRL-ablated layers with an ablation depth of 25 µm in the central and paracentral corneal region resulted in the identification of 4,454 proteins (Supplementary Table S1). Of these, 3,649 proteins were identified in at least three samples of each tissue layer and were used for further statistical testing (Supplementary Table S2). Both the individual samples and a collective analysis of all samples were evaluated.

**Figure 2.**
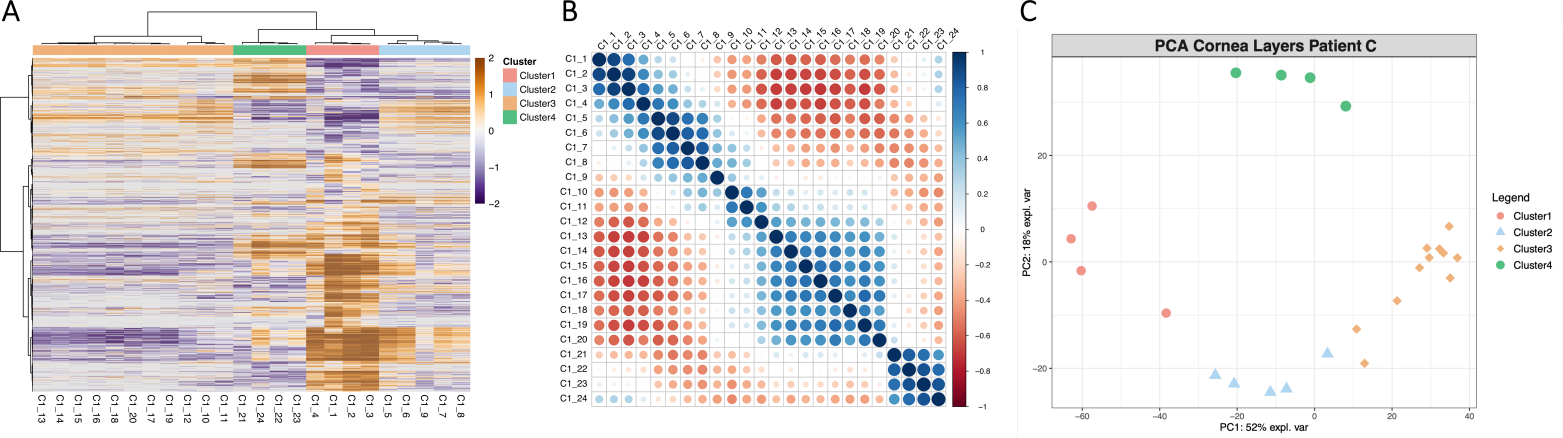
Data analysis results of individual C: **(A)** Heat map visualization of Pearson correlation-based unsupervised hierarchical clustering. **(B)** Heatmap visualization of Pearson correlation matrix. **(C)** Scatterplot visualization of nonlinear iterative partial least squares principal component analysis results.

Pearson correlation-based unsupervised hierarchical clustering across all corneal samples revealed a distinct separation between different corneal regions (Figs. 1A, 2), as well as notable inter-individual variability. Corneal regions characterized by proteomic profiling were assigned to clusters, designated according to their respective anatomical localization (“Epithelium”, “Sublayer”, “Stroma” and “Endothelium”). Nonlinear Iterative Partial Least Squares PCA displayed a pronounced separation of these clusters in each cornea. The analysis of all samples showed a clear differentiation of the stroma from other tissue layers, especially to the epithelium and endothelium, while few layers from the sublayer showed a mixed pattern between the epithelium and stroma.

Each cluster displayed different proteomic signatures. The relative abundances of various proteins were identified and systematically compared between each corneal cluster. In this way, associated specific functional processes within the cornea could be visualized.  GSEA was used to determine enrichment of GO terms in the categories BP, MF, and CC, based on the significance and expression patterns of all quantified proteins.

The results of Student's *t*-test have been incorporated into the data presented in Supplementary Table S2. All proteins that have been found to be significantly differentially abundant in the respective cluster (epithelium, sublayer, stroma, endothelium) are highlighted, and they can be filtered for *p*-value or *q*-value significance, as well as log_2_ difference (fold change). Supplementary Table S7 shows all proteins identified in at least one sample of only one cluster exclusively.

### Epithelium

**Figure 3.**
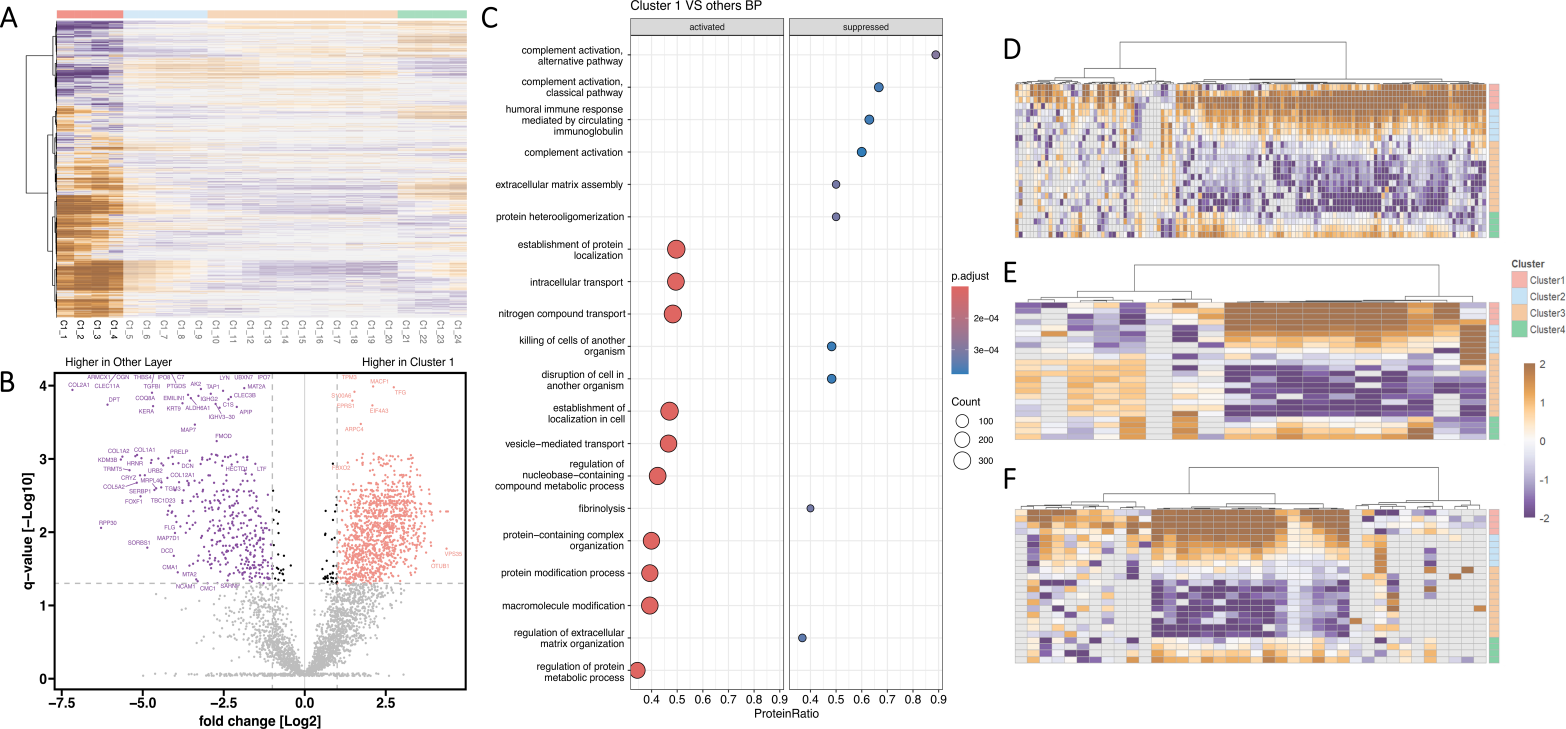
Epithelium: **(A)** Heat Map visualization of Pearson correlation-based unsupervised hierarchical clustering, highlighting the protein pattern for cluster 1 (assigned as Epithelium) in individual C. **(B)** Volcano plot visualization with significantly differentially abundant proteins displayed in *purple* (*q*-value < 0.05; fold change ≤ 2) and *red* (*q*-value < 0.05; fold change ≥ 2); **(C)** Gene set enrichment analysis plot for significantly abundant proteins (BP, biological processes); **(D–F)** Heat maps of highly abundant gene ontology biological process (GOBP): **(D)** cytoplasmic translation; **(E)** cell-cell recognition; **(F)** DNA biosynthetic process.

The distinct spatial proteomic region, which we annotated as Epithelium (Cluster 1), was identified based on its marked separation from surrounding clusters and characterized by a high abundance of proteins involved in metabolic and biosynthetic processes (Fig. 3A). Volcano plot analysis identified a large number of proteins with significantly higher abundance in the Epithelium compared to other corneal regions (Fig. 3B). Supplementary Table S3 lists all proteins identified in at least one sample in the defined epithelium, whereas Supplementary Table S8 shows the GSEA with the results of the *t*-test epithelium versus the other clusters.

This cluster showed high abundances of proteins involved in metabolic processes such as cytoplasmic translation (Figs. 3C, 3D), intracellular protein transport, vesicle-mediated trafficking, and posttranslational protein modification (Fig. 3C). The high translational output of the epithelium was demonstrated by the increased abundance of proteins involved in ribosomal biogenesis and RNA processing (Figs. 3C, 6). Furthermore, elevated signaling pathways of intercellular communication and structural epithelial integrity were observed via cell-cell recognition processes (Fig. 3E), and increased proliferative activity was indicated by DNA biosynthesis. Various proteins involved in nucleotide binding (including purine nucleotide binding, nucleoside binding, and adenyl ribonucleotide binding) have been shown to participate in signal transduction, barrier function, and RNA synthesis and processing (Fig. 6A). Their localization was enriched in metabolically active organelles (such as the outer endoplasmic reticulum), but they were also present in vesicles, cell junctions, and synaptic compartments (Fig. 6B). Immunogenic processes such as complement activation, immunoglobulin-mediated activation, and extracellular matrix assembly were underrepresented. In summary, various biological processes were demonstrated that contribute to the high regenerative capacity of the Epithelium and its role as a barrier interface.

In contrast to the clear proteomic differentiation among the other clusters, the sublayer showed only limited demarcation (Supplementary Table S4: all proteins identified in at least one sample in the sublayer, Supplementary Table S9: GSEA results of the *t*-test sublayer versus other clusters). Instead, it exhibited proteomic features characteristic of a composite structure between the epithelium and the stroma. Compared to the other clusters, there was an increased abundance of proteins involved in telomere organization and RNA binding. In addition to collagen synthesis, some metabolic pathways in response to cellular stress were enriched (Supplementary Figs. S1, S2).

### Stroma

**Figure 4.**
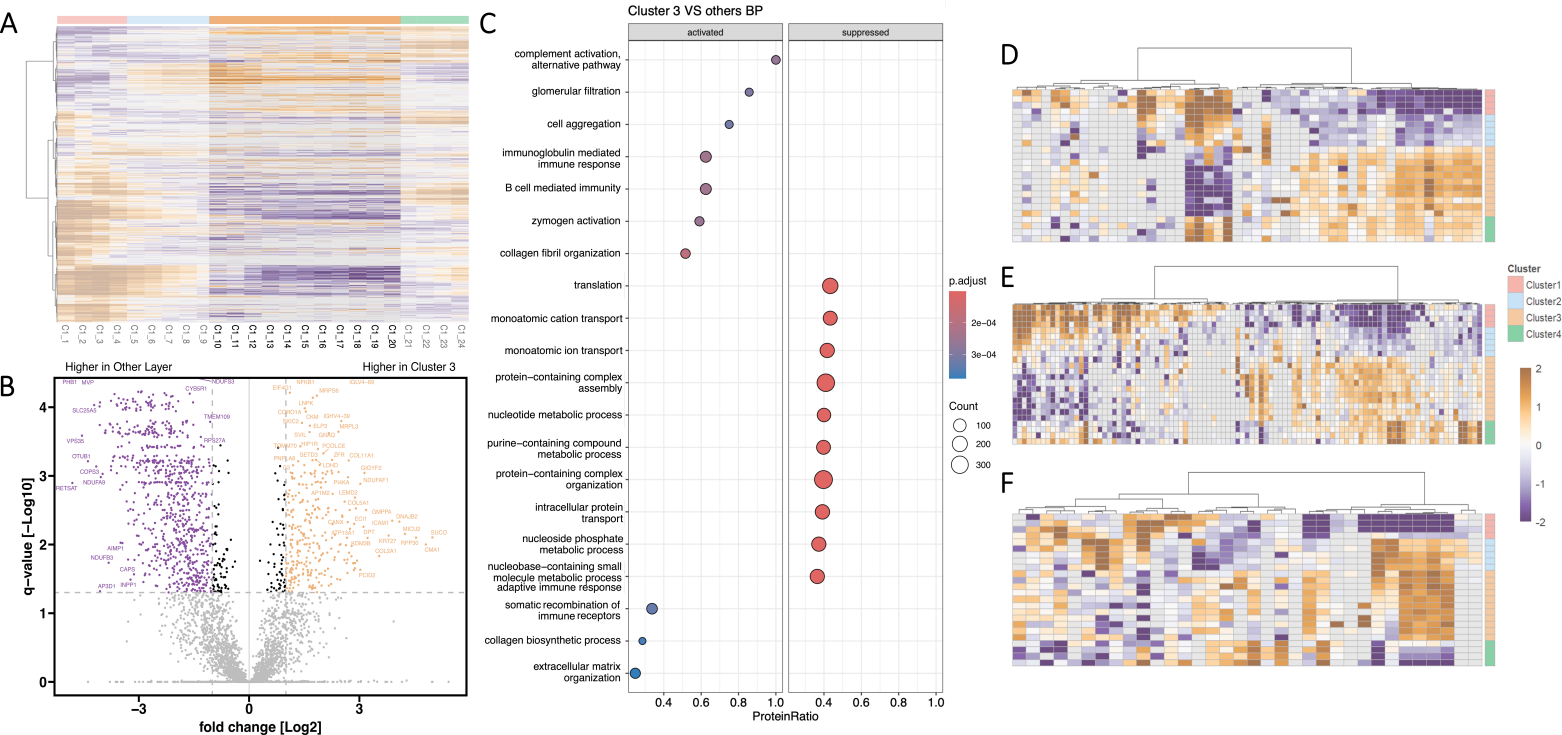
Stroma: **(A)** Heat map visualization of Pearson correlation-based unsupervised hierarchical clustering, highlighting the protein pattern for cluster 3 (assigned as stroma) in individual C. **(B)** Volcano plot visualization with significantly differentially abundant proteins displayed in *purple* (*q*-value < 0.05; fold change ≤ 2) and *yellow* (*q*-value < 0.05; fold change ≥ 2). **(C)** Gene set enrichment analysis plot for significantly abundant proteins (BP, biological processes). **(D–F)** Heat maps of highly abundant gene ontology biological process (GOBP): **(D)** immunoglobulin production; **(E)** leukocyte-mediated immunity; **(F)** collagen metabolic process.

The stroma (Cluster 3), the most voluminous layer, displayed a proteomic profile clearly distinct from that of the other layers (Fig. 4A). A large set of differentially abundant proteins was identified within this region (Fig. 4B). Supplementary Table S5 contains all proteins detected in at least one stromal sample, and Supplementary Table S10 provides the GSEA results based on the *t*-test comparing the stroma with the remaining layers. The stromal proteome was dominated by proteins involved in extracellular matrix organization, especially in collagen fibril production and assembly, whereas metabolic proteins were comparatively underrepresented (Figs. 4C, 4D). CC annotations revealed a pronounced enrichment of extracellular matrix-associated structures, including fibrillar and banded collagen trimers, as well as collagen type XI. These structural features were functionally accompanied by molecular functions such as proteoglycan binding and matrix components conferring tensile strength and resistance to compression, reflecting the biomechanical specialization of the corneal stroma. Higher abundances of proteins of the Golgi lumen were detected, corresponding to the many required post-translational modifications of the extracellular matrix (ECM) (Figs. 6A, 6B). A distinct increased abundance of immunoglobulins and proteins generally involved in the immune response could be visualized in the stromal region (Figs. 4C, 4E, 4F). Consistent with these findings, the molecular function and CC annotations of this cluster revealed an enrichment of antigen-binding proteins, immunoglobulins, and mediators of both humoral and innate immune pathways (Figs. 6A, 6B).

### Endothelium

**Figure 5.**
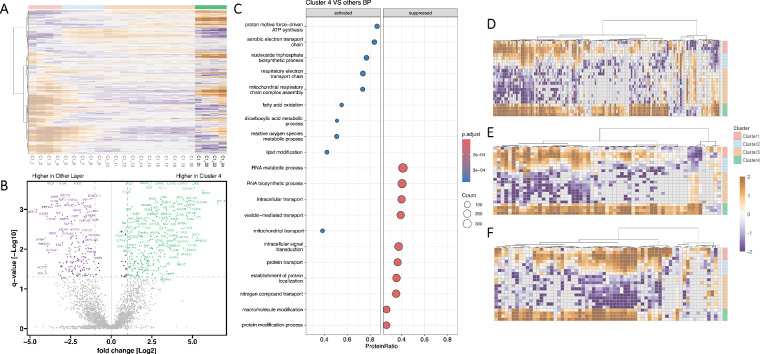
Endothelium: **(A)** Heat map visualization of Pearson correlation-based unsupervised hierarchical clustering, highlighting the protein pattern for cluster 4 (assigned as Endothelium) in individual C. **(B)** Volcano plot visualization with significantly differentially abundant proteins displayed in *purple* (*q*-value < 0.05; fold change ≤ 2) and *green* (*q*-value < 0.05; fold change ≥ 2). **(C)** Gene set enrichment analysis plot for significantly abundant proteins (BP, biological processes). **(D–F)** Heat maps of highly abundant gene ontology biological process (GOBP): **(D)** cellular respiration; **(E)** ATP biosynthetic process; **(F)** proton transmembrane transport.

The endothelium (Cluster 4) was resolved as a discrete proteomic layer, distinguishable from the adjacent stromal compartment (Fig. 5A). Numerous significantly altered protein abundances were detected within this compartment (Fig. 5B). Supplementary Table S6 lists proteins detected in the endothelium, and Supplementary Table S11 presents the endothelium versus other clusters *t*-test GSEA results.

**Figure 6.**
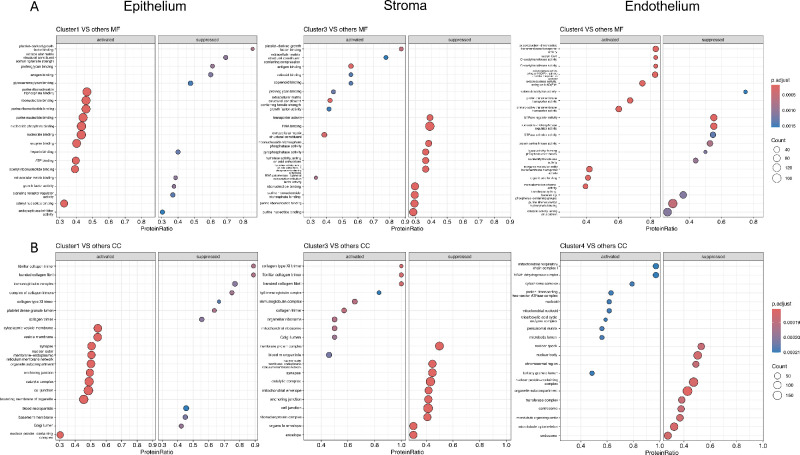
Gene set enrichment plot for *t*-test significantly abundant proteins: **(A)** Molecular Function (MF) and **(B)** cellular components.

In the cluster, proteins involved in energy metabolism were significantly higher abundant (Fig. 5C). This was particularly evident in mitochondrial metabolic pathways, including oxidative phosphorylation and ATP synthesis. Compared to the other clusters, there were elevated levels of mitochondria-localized proteins that generate ATP to support endothelial pumping activity and cellular homeostasis (Figs. 5C, 5D, 6B). Additionally, there was an increased abundance of energy-dependent transmembrane transport proteins for active transport processes. (Figs. 5C, 5F, 6B). Conversely, some biological processes, such as protein modification processes, were suppressed in the endothelium compared to other layers (Fig. 5C). In summary, this cluster exhibited a selective enrichment of energy-generating processes and transport pathways, reflecting the molecular basis of the endothelium's unique physiological role in preserving corneal clarity.

Many proteins showed depth-dependent abundance gradients across the cornea, rather than distinct demarcation at anatomical layer border. Epithelial structural and adhesion-associated factors progressively decreased with depth, while stromal extracellular matrix components peaked within the central layers. Proteins linked to endothelial matrix organization, ion transport, and metabolic activity showed a strong increase toward the innermost layers.

## Discussion

In this study, three-dimensional spatial proteomics of consecutive tissue layers of the human cornea was performed. This enables the precise mapping of the spatial distribution of various proteins and biological processes within the cornea (Fig. 1A).

The method of NIRL-based spatial sampling has already been applied and validated by our research group in other tissues, such as cerebral and intestinal tissue.^25^^,^^27^^,^^29^ In addition to providing spatial resolution, this method offers advantages such as the ability to resolve the proteome on a miniaturized scale and the elimination of the need for extensive tissue homogenization and requires only minimal additional steps for proteome extraction.^26^^,^^27^ By enabling spatially resolved proteomic analysis, this method provides new insights into the organization of biological processes and interactions within tissues—a perspective that has previously not been accessible in corneal research.

With this method, a total of 4,454 proteins in five corneas could be identified. To our knowledge, this is the second most comprehensive proteome analysis of the human cornea to date. Subbannayya et al.^14^ successfully identified 4,824 proteins by LC-MS/MS analysis of entire corneas from three individuals. In contrast, the NIRL-based approach used in our study allows for a spatially resolved, layer-specific analysis within a limited volume of the central cornea. Importantly, peripheral regions, particularly the limbal zone housing limbal stem cells, were not included in the present analysis. These areas likely harbor additional proteins that are absent in the central cornea.^30^^–^^32^ It is assumed that our analysis therefore provides a precise representation of the proteome within the central corneal region.

A separation into different clusters corresponding to anatomical structures within the cornea was shown. These clusters showed a high level of distinguishability within individuals. In particular, the proteomic distinction between the stroma, which is rich in organized collagen fibrils with just sparsely distributed keratocytes, and the cell-dense epithelial and endothelial layers was clearly observable. Furthermore, they exhibited biologically individual patterns that correspond to their anatomical localization as confirmed by OCT visualization. The distinct characteristics of the individual clusters not only align with findings from previous studies but also enhance our understanding of the proteome and the biological functions of the corneal layers.^15^^,^^19^^,^^33^^,^^34^

It could also be revealed that more substructures than previously assumed are required to properly describe the cornea and that the proteomic differences vary gradually throughout the layers. Although specific biological pathways are associated within one corneal layer, the abundance of the involved proteins is rather depicted as a gradient throughout the layers.

Matching its role as the outermost protective layer of the cornea, the rapidly replenishing cellular epithelium showed high abundances of enzymes participating in various metabolic pathways. As previously demonstrated, high levels of different proteins were involved in transcription, cytoplasmic translation and in cell maintenance of functional proteins.^19^ These findings align with the GSEA results observed in our study. The observation of high proportions of intracellular and extracellular vesicles suggests dynamic vesicular trafficking and active intracellular communication for regulating corneal wound repair and infection states.^11^ To form a strong barrier that prevents the penetration of pathogens into the deeper corneal layers, strong anchoring cell junctions are required, for which associated proteins have also been detected in high abundance in the epithelium cluster. The extracellular matrix proteins of the cell-dense epithelial layer were proportionally less pronounced than in the stroma. The epithelium is extensively innervated by free nerve endings, which contribute to its heightened sensitivity to physical stimuli.^35^^,^^36^ Proteins located in synapses could also be identified, which were not displayed accordingly in any other cluster.

According to the histological structure of the stroma, significantly higher abundances of ECM proteins, including especially fibrillar and banded collagen trimers, along with proteins that preserve structural integrity, could be identified in this cluster. Except for collagen metabolic processes, only a low abundance of other metabolic proteins was observed within the stromal cluster's limited cellular content. It was revealed that the previously reported high overall abundances of the proteins keratocan and lumican were more pronounced in the stroma and sublayer than in the epithelium and endothelium.^14^

Of particular interest is the significantly higher abundance of immune proteins in the stromal layer. The identified proteins included several immunoglobulin components, such as the heavy chains of immunoglobulins G, M, and A, as well as the variable and constant regions of the light chains λ (lambda) and κ (kappa). Moreover, multiple proteins of the complement cascade (C1, C3, C4B, C5, C7, Complement Factor H and B), together with Transforming Growth Factor Beta Induced (TGFBI) and Interferon Regulatory Factor 3 (IRF3), were present. The cornea possesses an inherent immune privilege to preserve its characteristic functions. In general, the cornea's complex immune defense mechanisms are designed to prevent excess inflammation, as severe inflammation could impair clear vision.^37^^–^^39^ There are fewer antigen-presenting cells in the central cornea and a lower concentration of factors for the humoral response than in the periphery to obtain the corneal transparency.^40^ In our proteome analysis, a higher abundance of immunoglobulins (IgG, IgM and IgA) was detected in the stroma. Surprisingly, proteins of the complement system also showed an increased abundance, suggesting a layer-specific immune preparedness. Unexpectedly, we found a significantly lower abundance of proteins involved in the classical pathway of complement activation in the epithelial and sublayer cluster. However, immune responses in the subepithelial region also frequently present subclinically.

In literature, high levels of IgG and IgA immunoglobulins were detected within the cornea, while IgM could not be found in the central region.^41^ It has already been described that proteomes of the complement system and immunoglobulins are among the most abundant proteins of the cornea.^14^^,^^17^^,^^19^ Meade et al.^19^ assumed an approximately even distribution of immune-related proteins across all corneal layers, with the main proteins identified as immunoglobulins. In contrast, our data suggest a stromal predominance of immunoglobulins. Further studies with functional validation are needed to demonstrate the localization and relative ratio of immunoglobulins within the cornea and the immune response process of the cornea.

Gene ontology biological process analysis of the endothelial cluster highlighted its role in maintaining corneal transparency via active water transport from the cornea to the anterior chamber.^8^ Correspondingly, a comparatively obvious increased abundance of active transmembrane transporters and mitochondrial proteins was observed within the endothelial layers. Highest normalized enrichment score included cellular respiration, oxidative phosphorylation and energy derivation by oxidation of organic compounds. These results can complement former proteomic studies that have partially focused on the proteomic integrity of endothelium and Descemet's membrane and thus also strongly on the ECM of this region.^19^^,^^21^^,^^22^^,^^42^

The respective basement membranes as for Bowman's membrane and Descemet's membrane of the cornea could not be effectively captured, likely because of the thickness of the membranes and the resolution limits of the laser ablation. Nevertheless, the subepithelial region displayed transitional abundance peaks, consistent with matrix remodeling at the interface of Bowman's layer and the anterior stroma.

Interestingly, there were subtle indications of a confined subepithelial region exhibiting a slightly distinct structural organization compared to the surrounding stromal tissue which was named “sublayer.” However, the demarcation between this region and the stromal cluster was not clearly defined in all analyses; therefore, it was not delineated with strict criteria. The proteomic characteristics of this suggested cluster, although not representing a clearly defined anatomical structure in the strict sense, revealed a gradual proteomic transition between the epithelium and the stroma. The anterior part of the corneal stroma contains a higher density and larger volume of highly active stromal keratocytes in contrast to the more organized posterior stroma.^43^^,^^44^ Indications of an involvement in wound response processes were observed. Telomere organization is one of the highest normalized enrichment score. For cells involved in wound healing maintaining telomere integrity is critical for optimal tissue repair.^45^ This finding is of particular relevance, because post-surgical corneal haze after photorefractive procedures is frequently localized to this region.^44^^,^^46^

Limitations of our study include the age of the donors and the number of corneas examined. However, we assume the sample size to be appropriate for the methodological aim to establish a workflow for spatially resolved corneal proteomics. Given the bradytrophic structure of the cornea and its relatively slow metabolic turnover, we consider it likely that our findings remain valid despite the advanced donor age. In future studies, it might also be beneficial to include functional validation assays (e.g., based on immunofluorescence microscopy).

Future studies aim to compare the spatial proteome of healthy corneas with that of corneal disorders such as keratoconus to facilitate the spatial localization of pathological molecular processes. This could improve the understanding of molecular and cellular pathomechanisms of various diseases and could take us one step further to the development of new therapeutic strategies.

Summarizing, our findings offer proteomic insights into key biological processes in the cornea, including immune response, wound healing and corneal homeostasis. Additionally, they provide a possibility for mapping the abundance of pharmacological target molecules, which could enhance the understanding of therapeutic mechanisms and drug delivery strategies.

## Supplementary Material

Supplement 1

Supplement 2

## References

[1] Blackburn BJ, Jenkins MW, Rollins AM, Dupps WJ. A review of structural and biomechanical changes in the cornea in aging, disease, and photochemical crosslinking. *Front Bioeng Biotechnol*. 2019; 7: 66. 31019909 10.3389/fbioe.2019.00066 PMC6459081

[2] Chowdhury DPH, Shah BH. Basics of anatomy and physiology of cornea. *Acta Scientific Ophthalmol*. 2021; 4: 2582–3191.

[3] Tidke SC, Tidake P. A review of corneal blindness: causes and management. *Cureus*. 2022; 14(10): e30097. 36381769 10.7759/cureus.30097 PMC9643016

[4] Flaxman SR, Bourne RRA, Resnikoff S, et al. Global causes of blindness and distance vision impairment 1990–2020: a systematic review and meta-analysis. *Lancet Glob Health*. 2017; 5(12): e1221–e1234. 29032195 10.1016/S2214-109X(17)30393-5

[5] Kate A, Basu S. Corneal blindness in the developing world: the role of prevention strategies. *F1000Res*. 2024; 12: 1309. 38618022 10.12688/f1000research.141037.2 PMC11009612

[6] Ting DSJ, Ho CS, Deshmukh R, Said DG, Dua HS. Infectious keratitis: an update on epidemiology, causative microorganisms, risk factors, and antimicrobial resistance. *Eye*. 2021; 35: 1084–1101. 33414529 10.1038/s41433-020-01339-3 PMC8102486

[7] Kumar A, Yun H, Funderburgh ML, Du Y. Regenerative therapy for the cornea. *Prog Retin Eye Res*. 2022; 87: 101011. 34530154 10.1016/j.preteyeres.2021.101011 PMC8918435

[8] Rates ERD, Almeida CD, Costa E de PF, Farias RJ de M, Santos-Oliveira R, Alencar LMR. Layer-by-layer investigation of ultrastructures and biomechanics of human cornea. *Int J Mol Sci*. 2022 ; 23: 7833. 35887181 10.3390/ijms23147833 PMC9317547

[9] Cui YH, Liu Q, Xu ZY, Li JH, Hu ZX, Li MJ, et al. Quantitative proteomic analysis of human corneal epithelial cells infected with HSV-1. *Exp Eye Res*. 2019; 185: 107664. 31085182 10.1016/j.exer.2019.05.004

[10] Moon CE, Kim CH, Jung JH, et al. Integrated analysis of transcriptome and proteome of the human cornea and aqueous humor reveal novel biomarkers for corneal endothelial cell dysfunction. *Int J Mol Sci*. 2023; 24: 15354. 37895034 10.3390/ijms242015354 PMC10607268

[11] Yeung V, Boychev N, Kanu LN, et al. Proteomic characterization of corneal epithelial and stromal cell-derived extracellular vesicles. *Int J Mol Sci*. 2024; 25: 10338. 39408670 10.3390/ijms251910338 PMC11477500

[12] Galiacy SD, Froment C, Mouton-Barbosa E, et al. Deeper in the human cornea proteome using nanoLC-Orbitrap MS/MS: an improvement for future studies on cornea homeostasis and pathophysiology. *J Proteomics*. 2011; 75: 81–92. 21989269 10.1016/j.jprot.2011.09.020

[13] Karring H, Thøgersen IB, Klintworth GK, Møller-Pedersen T, Enghild JJ. The human cornea proteome: Bioinformatic analyses indicate import of plasma proteins into the cornea. *Molecular Vision*. 2006; 12: 451–460. 16710169

[14] Subbannayya Y, Pinto SM, Mohanty V, Dagamajalu S, Prasad TSK, Murthy KR. What makes cornea immunologically unique and privileged? Mechanistic clues from a high-resolution proteomic landscape of the human cornea. *Omics*. 2020; 24: 129–139. 32125911 10.1089/omi.2019.0190

[15] Nishtala K, Panigrahi T, Shetty R, et al. Quantitative proteomics reveals molecular network driving stromal cell differentiation: implications for corneal wound healing. *Int J Mol Sci*. 2022; 23: 2572. 35269714 10.3390/ijms23052572 PMC8910342

[16] Shinde V, Hu N, Renuse S, et al. Mapping keratoconus molecular substrates by multiplexed high-resolution proteomics of unpooled corneas. *Omics*. 2019; 23: 583–597. 31651220 10.1089/omi.2019.0143 PMC6857467

[17] Dyrlund TF, Poulsen ET, Scavenius C, et al. Human cornea proteome: Identification and quantitation of the proteins of the three main layers including epithelium, stroma, and endothelium. *J Proteome Res*. 2012; 11: 4231–4239. 22698189 10.1021/pr300358k PMC3411198

[18] Karring H, Thøgersen IB, Klintworth GK, Møller-Pedersen T, Enghild JJ. A dataset of human cornea proteins identified by peptide mass fingerprinting and tandem mass spectrometry. *Mol Cell Proteomics*. 2005; 4: 1406–1408. 15911533 10.1074/mcp.D500003-MCP200

[19] Meade ML, Shiyanov P, Schlager JJ. Enhanced detection method for corneal protein identification using shotgun proteomics. *Proteome Sci*. 2009; 7: 23. 19563675 10.1186/1477-5956-7-23 PMC2711935

[20] Chaerkady R, Shao H, Scott SG, Pandey A, Jun AS, Chakravarti S. The keratoconus corneal proteome: loss of epithelial integrity and stromal degeneration. *J Proteomics*. 2013; 87: 122–131. 23727491 10.1016/j.jprot.2013.05.023 PMC3721369

[21] Nakagawa T, Okumura N, Ikegawa M, et al. Shotgun proteomics identification of proteins expressed in the Descemet's membrane of patients with Fuchs endothelial corneal dystrophy. *Sci Rep*. 2023; 13(1): 10401. 37369713 10.1038/s41598-023-37104-1 PMC10300001

[22] Poulsen ET, Dyrlund TF, Runager K, et al. Proteomics of fuchs endothelial corneal dystrophy support that the extracellular matrix of descemets membrane is disordered. *J Proteome Res*. 2014; 13: 4659–4667. 24846694 10.1021/pr500252r PMC4227554

[23] Bodzon-Kulakowska A, Bierczynska-Krzysik A, Dylag T, et al. Methods for samples preparation in proteomic research. *J Chromatogr B Analyt Technol Biomed Life Sci*. 2007; 849(1–2): 1–31. 10.1016/j.jchromb.2006.10.040 17113834

[24] Goldberg S. Mechanical/physical methods of cell disruption and tissue homogenization. *Methods Mol Biol*. 2008; 424: 3–22. 18369848 10.1007/978-1-60327-064-9_1

[25] Voss H, Moritz M, Pelczar P, et al. Tissue sampling and homogenization with NIRL enables spatially resolved cell layer specific proteomic analysis of the murine intestine. *Int J Mol Sci*. 2022; 23: 6132. 35682811 10.3390/ijms23116132 PMC9181169

[26] Hahn J, Moritz M, Voss H, Pelczar P, Huber S, Schlüter H. Tissue sampling and homogenization in the sub-microliter scale with a nanosecond infrared laser (NIRL) for mass spectrometric proteomics. *Int J Mol Sci*. 2021; 22(19): 10833. 34639174 10.3390/ijms221910833 PMC8509473

[27] Navolić J, Moritz M, Voss H, et al. Direct 3D sampling of the embryonic mouse head: layer-wise nanosecond infrared laser (NIRL) ablation from scalp to cortex for spatially resolved proteomics. *Anal Chem*. 2023; 95: 17220–17227. 37956982 10.1021/acs.analchem.3c02637 PMC10688223

[28] Subramanian A, Tamayo P, Mootha VK, et al. Gene set enrichment analysis: a knowledge-based approach for interpreting genome-wide expression profiles. *Proc Natl Acad Sci USA*. 2005; 102: 15545–15550. 16199517 10.1073/pnas.0506580102 PMC1239896

[29] Navolić J, Hawass S, Moritz M, et al. Spatial proteomics reveals distinct protein patterns in cortical migration disorders caused by LIN28A overexpression and WNT activation. *Mol Cell Proteomics*. 2025; 24(9): 101037. 40680886 10.1016/j.mcpro.2025.101037 PMC12419088

[30] Honoré B, Vorum H. Proteomic analysis as a means to approach limbal stem cell biology in a search for stem cell markers. *Proteomics Clin Appl*. 2014; 8(3–4): 178–184. 24497450 10.1002/prca.201300049

[31] Meshko B, Volatier TLA, Hadrian K, et al. ABCB5+ limbal epithelial stem cells inhibit developmental but promote inflammatory (lymph) angiogenesis while preventing corneal inflammation. *Cells*. 2023; 12: 1731. 37443766 10.3390/cells12131731 PMC10341195

[32] Polisetti N, Sharaf L, Schlötzer-Schrehardt U, Schlunck G, Reinhard T. Efficient isolation and functional characterization of niche cells from human corneal limbus. *Int J Mol Sci*. 2022; 23: 2750. 35269891 10.3390/ijms23052750 PMC8911296

[33] Liu Y, Huang H, Sun G, et al. Gene expression profile of extracellular matrix and adhesion molecules in the human normal corneal stroma. *Curr Eye Res*. 2017; 42: 520–527. 27442190 10.1080/02713683.2016.1200099 PMC6011830

[34] Wu YF, Chang NW, Chu LA, et al. Single-cell transcriptomics reveals cellular heterogeneity and complex cell–cell communication networks in the mouse cornea. *Invest Ophthalmol Vis Sci*. 2023; 64(13): 5. 10.1167/iovs.64.13.5 PMC10565710 37792336

[35] Al-Aqaba MA, Dhillon VK, Mohammed I, Said DG, Dua HS. Corneal nerves in health and disease. *Prog Retin Eye Res*. 2019; 73: 100762. 31075321 10.1016/j.preteyeres.2019.05.003

[36] Stepp MA, Tadvalkar G, Hakh R, Pal-Ghosh S. Corneal epithelial cells function as surrogate Schwann cells for their sensory nerves. *Glia*. 2017; 65: 851–863. 27878997 10.1002/glia.23102 PMC5395310

[37] Hori J, Yamaguchi T, Keino H, Hamrah P, Maruyama K. Immune privilege in corneal transplantation. *Prog Retin Eye Res*. 2019; 72: 100758. 31014973 10.1016/j.preteyeres.2019.04.002

[38] Downie LE, Zhang X, Wu M, et al. Redefining the human corneal immune compartment using dynamic intravital imaging. *Proc Natl Acad Sci USA.* 2023; 120(31): e2217795120. 37487076 10.1073/pnas.2217795120 PMC10400993

[39] Liu J, Li Z. Resident innate immune cells in the cornea. *Front Immunol.* 2021; 12: 620284. 33717118 10.3389/fimmu.2021.620284 PMC7953153

[40] Ruiz-Lozano RE, Salan-Gomez M, Rodriguez-Garcia A, et al. Wessely corneal ring phenomenon: an unsolved pathophysiological dilemma. *Surv Ophthalmol*. 2023; 68: 713–727. 36882129 10.1016/j.survophthal.2023.02.009

[41] Allansmith MR, McClellan BH. Immunoglobulins in the human cornea. *Am J Ophthalmol*. 1975; 80: 123–132. 1155538 10.1016/0002-9394(75)90882-x

[42] Halfter W, Moes S, Halfter K, et al. The human Descemet's membrane and lens capsule: protein composition and biomechanical properties. *Exp Eye Res*. 2020; 201: 108326. 33147472 10.1016/j.exer.2020.108326

[43] Muller LJ, Pels L, Vrensen GFJM. Novel aspects of the ultrastructural organization of human corneal keratocytes. *Invest Ophthalmol Vis Sci*. 1995; 36: 2557–2567. 7499078

[44] Patel S V., McLaren JW, Hodge DO, Bourne WM. Normal human keratocyte density and corneal thickness measurement by using confocal microscopy in vivo. *Invest Ophthalmol Vis Sci*. 2001; 42: 333–339. 11157863

[45] Saito Y, Yamamoto S, Chikenji TS. Role of cellular senescence in inflammation and regeneration. *Inflamm Regen*. 2024; 44: 28. 38831382 10.1186/s41232-024-00342-5 PMC11145896

[46] Charpentier S, Keilani C, Maréchal M, et al. Corneal haze post photorefractive keratectomy. *J Fr Ophtalmol*. 2021; 44: 1425–1438. 34538661 10.1016/j.jfo.2021.05.006

