# Supplementary material for: Layer-Specific Proteomic Profiling of the Human Cornea Reveals Insights Into Structure and Biological Function
Source: Invest Ophthalmol Vis Sci. 2026 Jan 21;67(1):44. doi: 10.1167/iovs.67.1.44 (PMC12831156; doi:10.1167/iovs.67.1.44)
Supplement: Supplement 1 [file iovs-67-1-44_s001.pdf]

Supplementary Material

The GSEA results were visualized with the R package “enrichplot” and “DOSE” by applying the p-values and the protein ratios for each gene set.

Pearson correlation-based unsupervised hierarchical clustering with ward.D linkage was visualized with pheatmap package (version 1.0.10) in R software environment. Volcano plots were created with ggplot2 package (version 3.5.1) in R software environment. Correlation plots were visualized using the corplot package (version 0.95) and NIPALS-PCA was created using the mixOmics package (version 6.30.0) in R software environment.

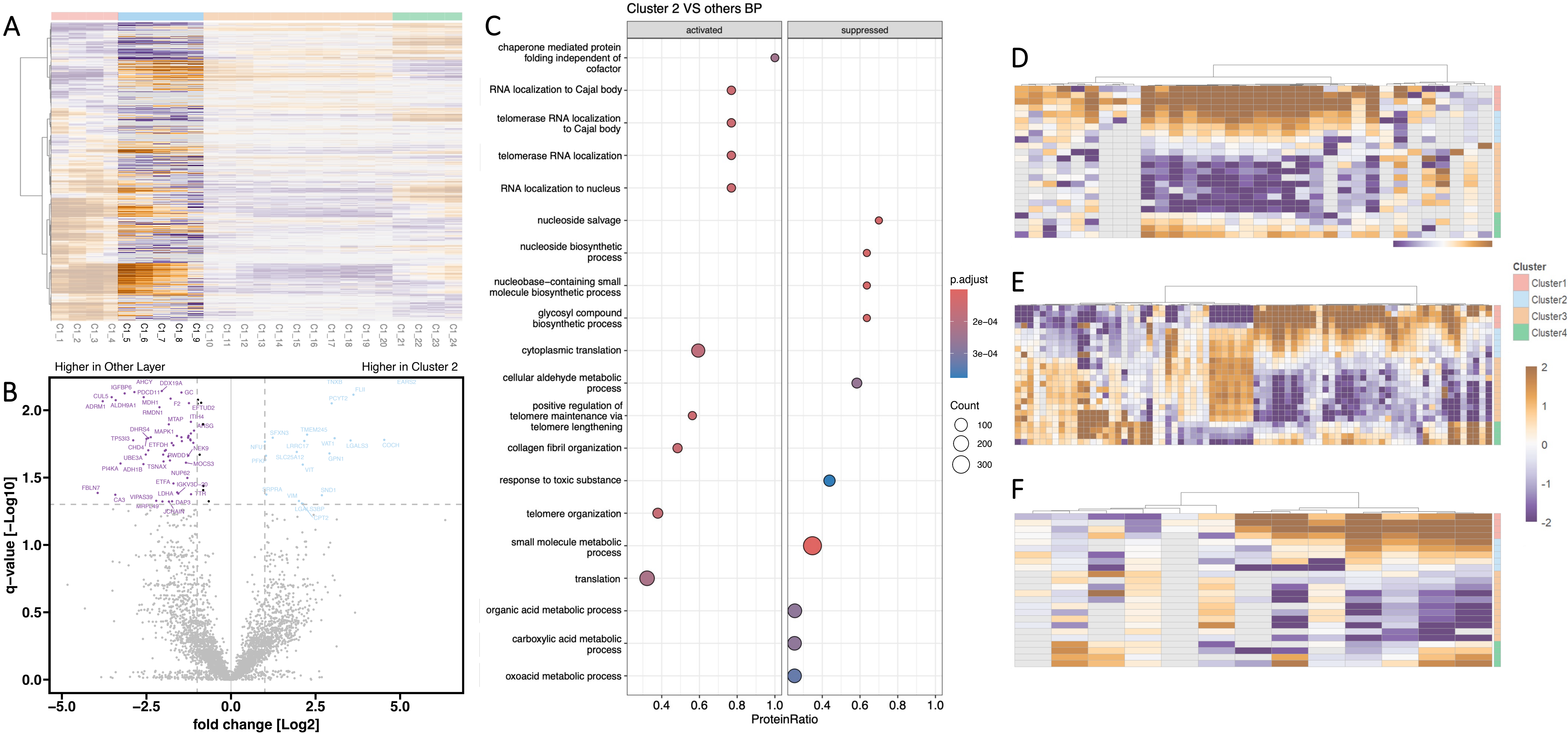

A
C1_1
C1_2
C1_3
C1_4
C1_5
C1_6
C1_7
C1_8
C1_9
C1_10
C1_11
C1_12
C1_13
C1_14
C1_15
C1_16
C1_17
C1_18
C1_19
C1_20
C1_21
C1_22
C1_23
C1_24
B
Higher in Other Layer
Higher in Cluster 2
q-value [-Log10]
fold change [Log2]
C
Cluster 2 VS others BP
activated
suppressed
chaperone mediated protein folding independent of cofactor
RNA localization to Cajal body
telomerase RNA localization to Cajal body
telomerase RNA localization
RNA localization to nucleus
nucleoside salvage
nucleoside biosynthetic process
nucleobase-containing small molecule biosynthetic process
glycosyl compound biosynthetic process
cytoplasmic translation
cellular aldehyde metabolic process
positive regulation of telomere maintenance via telomere lengthening
collagen fibril organization
response to toxic substance
telomere organization
small molecule metabolic process
translation
organic acid metabolic process
carboxylic acid metabolic process
oxoacid metabolic process
ProteinRatio
p.adjust
2e-04
3e-04
Count
100
200
300
D
E
F
Cluster
Cluster1
Cluster2
Cluster3
Cluster4

Supplementary Figure S1: Sublayer: A) Heat Map visualization of Pearson correlation-based unsupervised hierarchical clustering, highlighting the protein pattern for cluster 2 (assigned as Sublayer) in individual C; B) Volcano plot visualization with significantly differentially abundant proteins displayed in purple (q-value < 0.05; fold change ≤ 2) and blue (q-value < 0.05; fold change ≥ 2); C) Gene Set Enrichment Analysis Plot for significantly abundant proteins (BP - Biological processes); D)-F) Heat Maps of highly abundant Gene Ontology Biological Process (GOBP): D) RNA-templated DNA Biosynthetic Process; E) Regulation of Chromosome Organization; F) Positive Regulation of Epithelial Cell Differentiation

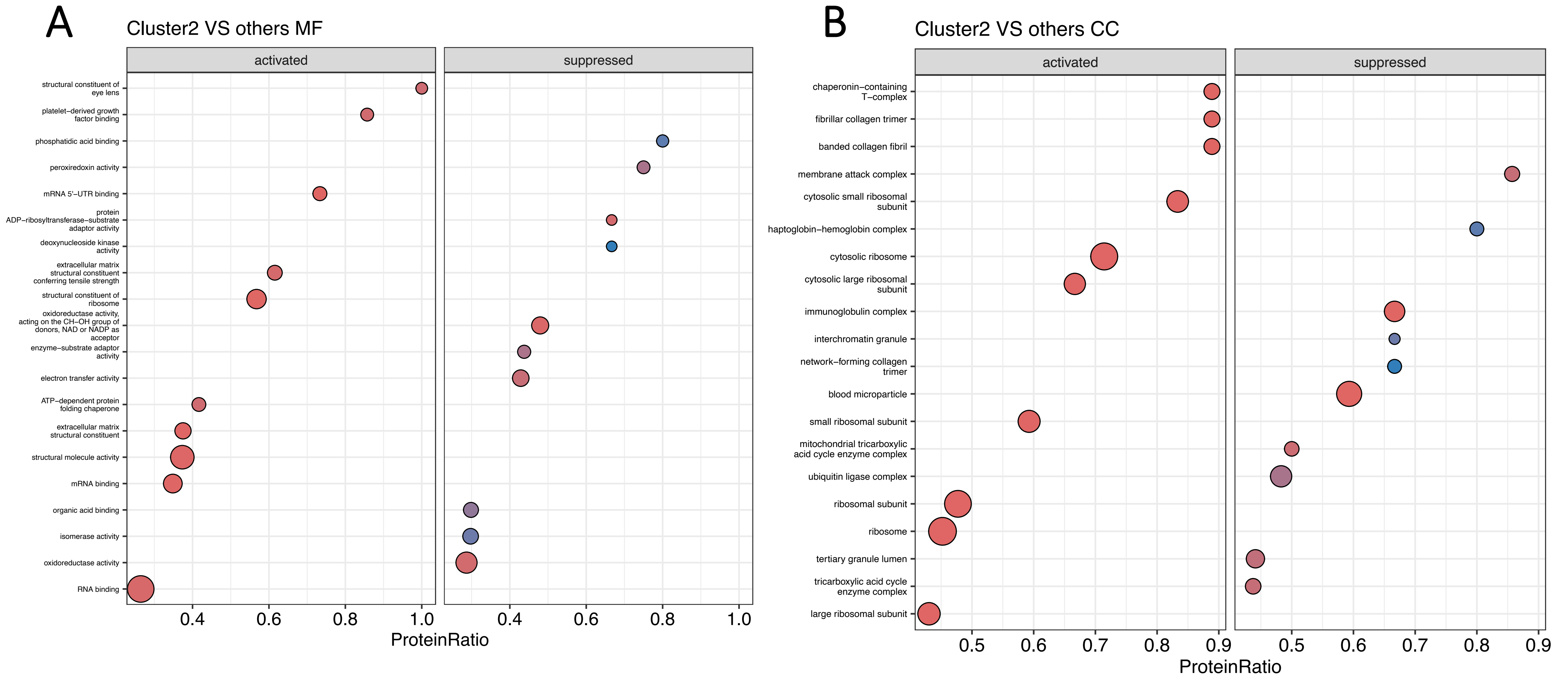

A
Cluster2 VS others MF
activated
suppressed
structural constituent of eye lens
platelet−derived growth factor binding
phosphatidic acid binding
peroxiredoxin activity
mRNA 5'−UTR binding
protein ADP−ribosyltransferase−substrate adaptor activity
deoxynucleoside kinase activity
extracellular matrix structural constituent conferring tensile strength
structural constituent of ribosome
oxidoreductase activity, acting on the CH−OH group of donors, NAD or NADP as acceptor
enzyme−substrate adaptor activity
electron transfer activity
ATP−dependent protein folding chaperone
extracellular matrix structural constituent
structural molecule activity
mRNA binding
organic acid binding
isomerase activity
oxidoreductase activity
RNA binding
0.4 0.6 0.8 1.0
ProteinRatio
B
Cluster2 VS others CC
chaperonin−containing T−complex
fibrillar collagen trimer
banded collagen fibril
membrane attack complex
cytosolic small ribosomal subunit
haptoglobin−hemoglobin complex
cytosolic ribosome
cytosolic large ribosomal subunit
immunoglobulin complex
interchromatin granule
network−forming collagen trimer
blood microparticle
small ribosomal subunit
mitochondrial tricarboxylic acid cycle enzyme complex
ubiquitin ligase complex
ribosomal subunit
ribosome
tertiary granule lumen
tricarboxylic acid cycle enzyme complex
large ribosomal subunit
0.5 0.6 0.7 0.8 0.9
ProteinRatio

Supplementary Figure S2: Sublayer; Gene Set Enrichment Plot for T-test significantly abundant proteins: A) Molecular Function (MF), B) Cellular Components
